# MiR-16-5p suppresses myofibroblast activation in systemic sclerosis by inhibiting NOTCH signaling

**DOI:** 10.18632/aging.202308

**Published:** 2020-12-19

**Authors:** Qicen Yao, Yixi Xing, Zaiyan Wang, Jin Liang, Qianqi Lin, Meiqiong Huang, Yiling Chen, Bo Lin, Xiayu Xu, Weifei Chen

**Affiliations:** 1 Department of Rheumatology and Immunology, The Second Affiliated Hospital of Hainan Medical University, Haikou 570311, Hainan Province, China; 2 Department of Respiratory Medicine, The affiliated Zhoupu Hospital, Shanghai University of Medicine and Health Sciences, Shanghai 201318, China; 3 Department of Pharmacy, The Second Affiliated Hospital of Hainan Medical University, Haikou 570311, Hainan Province, China

**Keywords:** SSc, miRNA, myofibroblast, NOTCH

## Abstract

Systemic sclerosis (SSc) is a prototypic fibrotic disease characterized by localized or diffuse skin thickening and fibrosis. Tissue fibrosis is driven by myofibroblasts, and factors affecting myofibroblast activation may also be involved in the development of SSc. In this study, we examined molecular mechanisms underlying SSc by focusing on myofibroblast activation processes. Bioinformatics analysis conducted to identify differentially expressed miRNAs (DEMs) and genes (DEGs) revealed that microRNA-16-5p (miR-16-5p) was downregulated and NOTCH2 was upregulated in SSc patients. *In vitro* experiments confirmed that miR-16-5p was able to bind directly to NOTCH2 and inhibit myofibroblast activation. Moreover, miR-16-5p-dependent inhibition of NOTCH2 decreased collagen and α-SMA expression. MiR-16-5p downregulation and NOTCH2 upregulation was also confirmed *in vivo* in SSc patients, and NOTCH2 activation promoted fibrosis progression *in vitro*. These results indicate that miR-16-5p suppresses myofibroblast activation by suppressing NOTCH signaling.

## INTRODUCTION

Systemic sclerosis (SSc), a complex and heterogeneous connective tissue disease, is observed mainly in female patients. The main characteristics of SSc include immune abnormalities, microvasculopathy, excessive extracellular deposition, and progressive fibrosis of the skin and other internal organs [1]. Currently, the mechanisms underlying SSc remain largely unknown, although infection, immune activation by cancer, and dysbiosis might trigger SSc on the genetic level [2, 3]. In addition, serious complications of SSc, such as interstitial lung disease, pulmonary hypertension, and heart failure, can be life-threatening [4].

Fibroblasts play an important role in fibrosis and maintain profibrotic phenotypes *in vitro* when explanted from affected tissues, resulting in increased secretion of collagen, extra-cellular matrix proteins, and alpha-smooth muscle actin (α-SMA), a crucial marker of myofibroblasts [5]. Additional *in vitro* studies are needed to investigate the underlying molecular and epigenetic mechanisms related to fibrosis [6]. For example, a recent study demonstrated that transforming growth factor beta (TGF-β) played a key role in SSc by regulating fibrosis progression [7].

microRNAs (miRNAs), a type of small non-coding RNA molecule approximately 22 nucleotides in length, are present in plants, animals, and some viruses, and can both silence RNA expression and regulate gene expression post-transcriptionally [8]. miRNA expression has been associated with regulation of developmental processes and a variety of disease states [9]. Accumulating evidence suggests that miRNA plays a crucial role in many fibrotic conditions [10]. For example, miR-16-5p regulates epigenetic modification of the connective tissue and plays a key role in immune diseases [11]. miR-16-5p also modulates inflammatory cytokines and might be crucial for anti-inflammatory response [12].

It is well known that the NOTCH family of cell surface receptors is crucial for intercellular communication [13]. When activated by ligand binding, the NOTCH intracellular domain (NICD) is released, forms a transcriptional activator complex with RBPJ/RBPSUH, and activates genes in the enhancer of split locus [14]. It also affects the initiation of tissue differentiation, proliferation, and apoptotic programs [15]. It was previously demonstrated that NOTCH signaling is regulated by certain miRNAs, such as miR-164a, miR-34a, and miR-143-3p [16–18].

In this study, we examined miR-16-5p expression in SSc patients and found that miR-16-5p suppressed profibrotic activation of dermal fibroblasts *in vitro.* Furthermore, we show that this phenotype is driven by miR-16-5p-mediated NOTCH pathway suppression.

## RESULTS

### Identification of DEGs and DEMs

**Figure 1:**
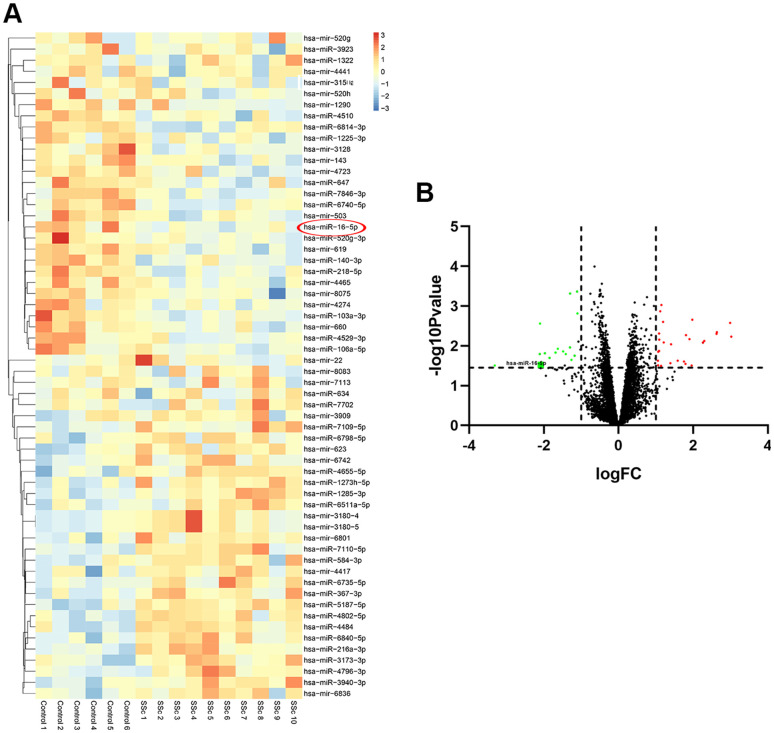
**Differentially expressed miRNAs in SSc patient samples compared to healthy controls.** (**A**) The heat map shows hierarchical clustering of differentially expressed miRNAs in SSc patients compared to healthy controls (n=30 per group) in the GSE137472 dataset. (**B**) The volcano plot shows differentially expressed miRNAs in SSc patients compared to healthy controls (n=30 per group) in the GSE137472 dataset.

**Figure 2:**
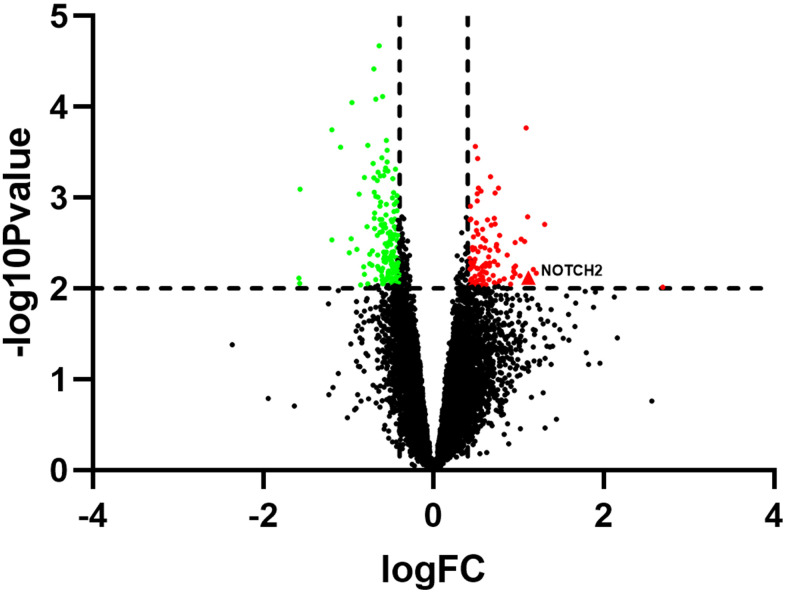
**Volcano plot showing differentially expressed genes in SSc patients compared to healthy controls (n=30 per group) in the GSE145120 dataset.**

The GSE137472 (miRNA) and GSE145120 (mRNA) gene expression profiles were obtained from the GEO database. The data were analyzed using the GEO2R online tool. A total of 28 upregulated and 29 downregulated DEMs, including miR-16-5p, were identified in SSc patients as compared to normal controls. 392 upregulated DEGs, including NOTCH2, and 348 downregulated DEGs were identified in SSc patients compared to controls. The top 30 up-regulated and top 30 down-regulated miRNAs and mRNAs are shown in Figures 1 and 2, respectively.

### miRNA-targeted gene prediction

**Figure 3:**
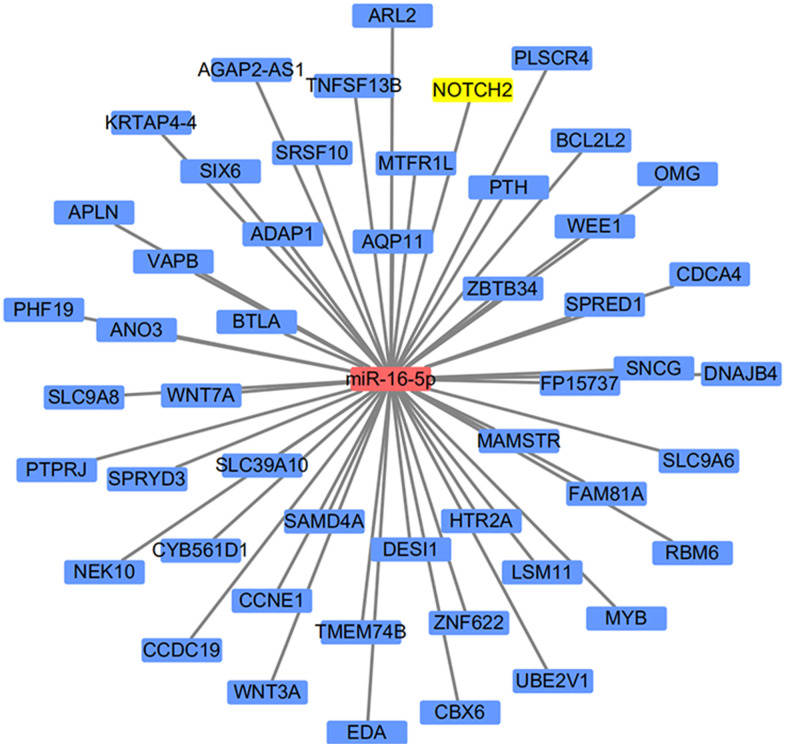
**Schematic diagram of the miRNA target prediction strategy.**

miR-16-5p, which was down-regulated in SSc, has been reported as an epigenetic regulator of the connective tissue as well as a key regulator in immune diseases [11]. We therefore searched TargetScanHiman to identify potential downstream targets of miR16-5p; 1515 potential targeted genes were identified. Interestingly, NOTCH2 was both listed as a potential target gene of miR-16-5p and was among the DEGs identified in SSc patients, and the Notch family of cell surface receptors is crucial for intercellular communication [13]. These results suggest that the effects of miR-16-5p may be in mediated in part by downstream NOTCH2 activity; *in vitro* and *in vivo* experiments supported this hypothesis. A schematic diagram of the target gene prediction process is shown in Figure 3.

### GO and KEGG enrichment analysis

**Figure 4:**
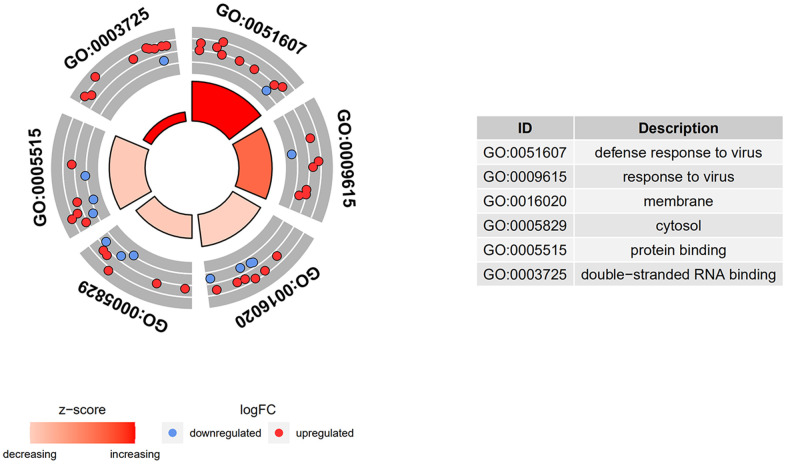
**GO and KEGG enrichment analysis of DEGs.** DEGs in SSc patients were significantly enriched in the following categories and pathways: in the biological process category, defense response to virus, response to virus, and type I interferon; in the cellular component category, membrane, cytosol, and Golgi apparatus; in the molecular function category, protein binding, double-stranded RNA binding, and phosphatase activity.

**Figure 5:**
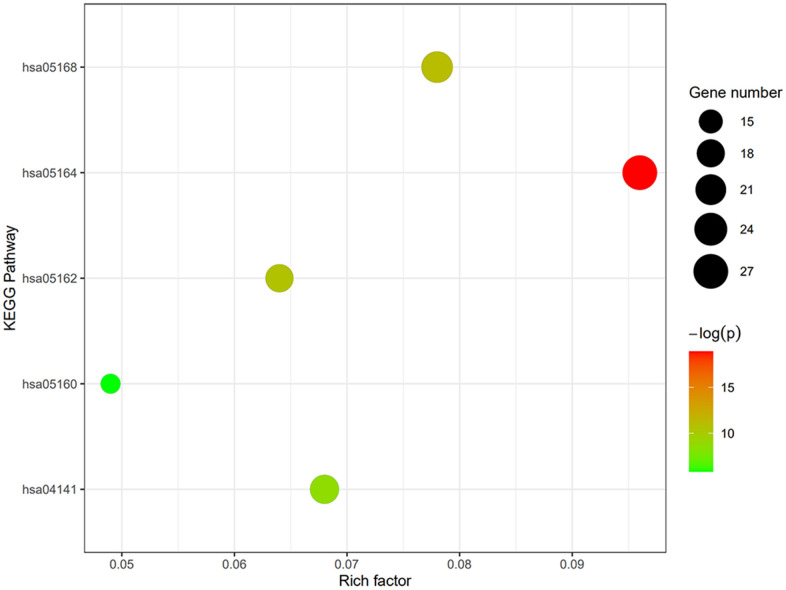
**Degree, betweenness, and closeness centrality of hub genes.** The top 5 KEGG pathways of DEGs in SSc patients were influenza A, herpes simplex infection, measles, protein processing in endoplasmic reticulum, and hepatitis C.

GO term analysis and KEGG pathway enrichment analyses were performed using the DAVID bioinformatics tool. DEGs in SSc patients were significantly enriched in the following categories and processes: in the biological process category, defense response to virus, response to virus, and type I interferon; in the cellular component category, membrane, cytosol, and Golgi apparatus; in the molecular function category, protein binding, double-stranded RNA binding, and phosphatase activity (Table 1 and Figure 4). The top 5 KEGG pathways associated with DEGs in SSc patients were influenza A, herpes simplex infection, measles, protein processing in endoplasmic reticulum, and hepatitis C (Table 2 and Figure 5).

**Table 1:** GO functional enrichment analysis of the genes in module.

**A, Biological process**			
**Term**	**Name**	**Count**	**P-value**
GO:0051607	defense response to virus	29	3.03E-11
GO:0009615	response to virus	22	9.79E-10
GO:0060337	type I interferon	17	1.57E-9
**B, Cellular component**			
**Term**	**Name**	**Count**	**P-value**
GO:0016020	membrane	134	2.02E-9
GO:0005829	cytosol	174	2.38E-7
GO:0005794	golgi apparatus	61	1.33E-6
**C, Molecular function**			
**Term**	**Name**	**Count**	**P-value**
GO:0005515	protein binding	408	2.74E-10
GO:0003725	double-stranded RNA binding	10	4.30E-4
GO:0016791	phosphatase activity	7	3.57E-3
GO: Gene Ontology.			

Top 2 terms were selected according to P-value when more than 2 terms enriched terms were identified in each category.

**Table 2:** KEGG pathway enrichment analysis of the genes in module.

**KEGG pathway**			
**Term**	**Name**	**Count**	**P-value**
Hsa05164	Influenza A	27	6.24E-9
Hsa05168	Herpes simplex infection	22	1.49E-5
hsa05162	Measles	18	2.46E-5
hsa04141	Protein processing in endoplasmic reticulum	19	1.62E-4
hsa05160	Hepatitis C	14	2.85E-3
KEGG, Kyoto Encyclopedia of Genes and Genomes.			

Top 5 terms were selected according to P-value when more than 5 terms enriched terms were identified in each category.

### Inhibition of miR-16-5p activates myofibroblasts by targeting NOTCH2

**Figure 6:**
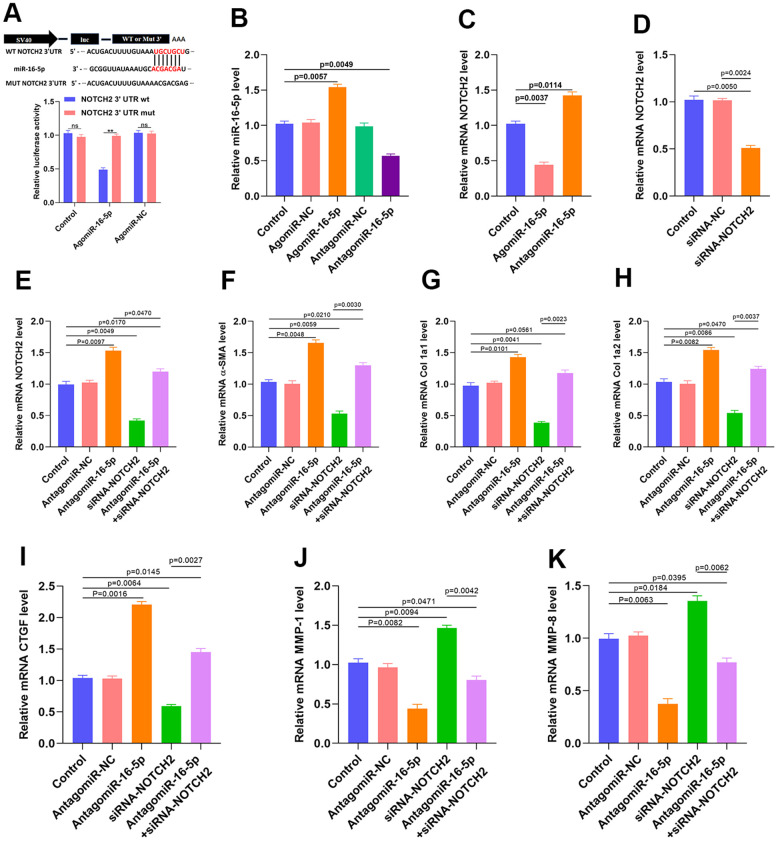
**Inhibition of miR-16-5p activates myofibroblasts.** (**A**) Luciferase assay results. (**B**) miR-16-5p transfection rate was measured by qRT-PCR. (**C**) NOTCH2 expression in the different groups. (**D**) siRNA transfection rate was measured by qRT-PCR. (**E**–**I**) Levels of collagen-related markers were measured by qRT-PCR after different treatments in HSFs. (**J**, **K**) MMP levels in the different groups were measured by qRT-PCR. Data are shown as means ± SDs of three independent experiments.

A luciferase assay performed to verify the association between miR-16-5p and NOTCH2 indicated that miR-16-5p specifically bound to the predicted NOTCH2 mRNA target region. Furthermore, miR-16-5p no longer reduced luciferase activity when a mutation was present in the predicted NOTCH2 target region (Figure 6A and Supplementary Figure 1). miR-16-5p agonist and antagonists were then transfected, and successful transfection was confirmed using qRT-PCR (Figure 6B). qRT-PCR and western blot results also demonstrated that NOTCH2 expression was clearly suppressed when miR-16-5p was upregulated (Figure 6C and Supplementary Figure 2). Small interfering RNA (siRNA) was then used to inhibit NOTCH2 expression in HSFs, and qRT-PCR indicated that the NOTCH2 siRNA transfection was successful (Figure 6D and Supplementary Figure 3). Furthermore, NOTCH2 siRNA partially reversed the miR-16-5p antagonist-induced increase in NOTCH2 levels (Figure 6E). We then examined the expression of profibrotic marker levels in fibroblasts expressing antagomiR-16-5p and NOTCH2 siRNA. Inhibition of miR-16-5p in HSFs increased levels of collagen type 1A1 (Col 1A1), 1A2 (Col 1A2), α-SMA, and connective tissue growth factor (CTGF) transcript (Figure 6F–6I). In addition, matrix metallopeptidase 1 (MMP-1) and matrix metallopeptidase 8 (MMP-8) expression decreased when miR-16-5p was inhibited, and NOTCH2 knockdown partially reversed that decrease (Figure 6J, 6K). Vimentin, Galectin-3, transforming growth factor-β (TGF-β), and collagen type1 expression and release were also investigated *in vitro*; those results are shown in Supplementary Figure 4.

### miR-16-5p and NOTCH2 expression in SSc patients and NOTCH2 overexpression-induced myofibroblast activation in HSFs

**Figure 7:**
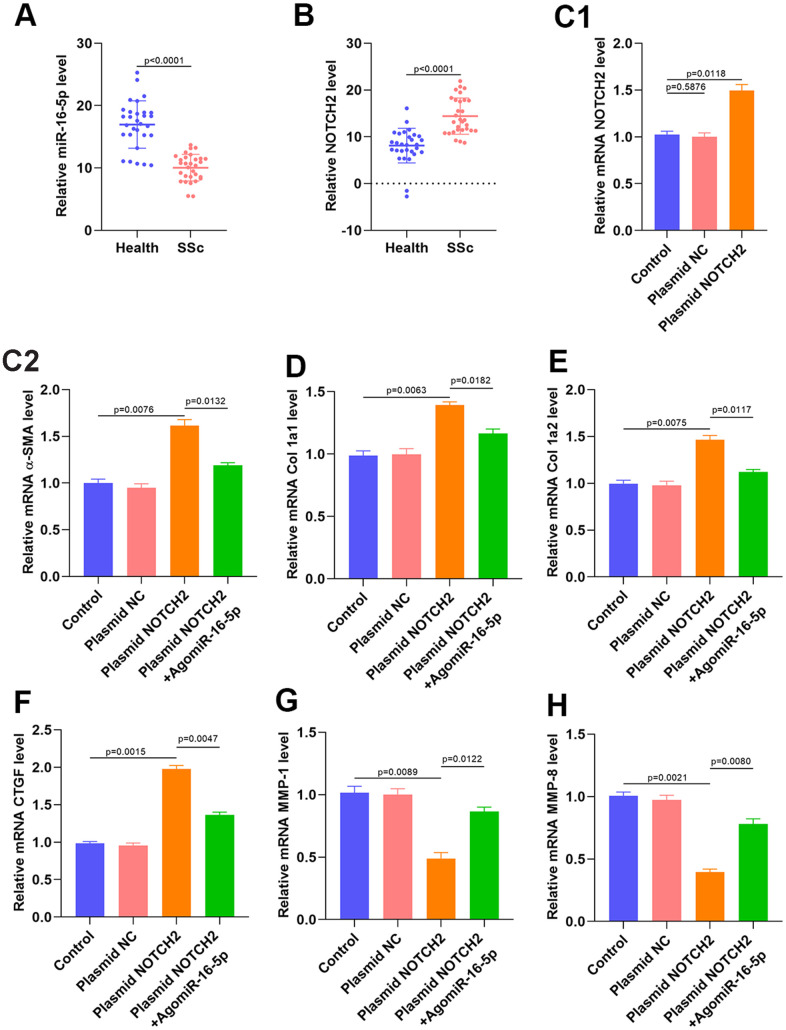
**NOTCH2 is overexpressed in SSc patients and promotes fibrosis *in vitro.*** (**A**, **B**) miR-16-5p and NOTCH2 expression in SSc patients and healthy controls (n=30 per group). (**C1**) The effects of NOTCH2 plasmid transfection on NOTCH2 levels in HSFs was assessed using qRT-PCR. (**C2**–**F**) Levels of collagen-related markers were measured by qRT-PCR analysis after different treatments in HSFs. (**G**–**H**) MMP levels in the different groups were measured by qRT-PCR. Data are shown as means ± SDs of three independent experiments.

qRT-PCR and western blot analyses of serum samples collected from SSc patients and healthy controls suggested that miR-16-5p was downregulated and NOTCH2 was upregulated in SSc patients (Figure 7A–7B and Supplementary Figures 5 and 6). Transfection of a NOTCH2 overexpression plasmid resulted in the expected increase in NOTCH2 levels in HSFs according to qRT-PCR and western blot analysis (Figure 7C and Supplementary Figure 7). We then measured the expression of profibrotic markers in NOTCH2 overexpressing fibroblasts. NOTCH2 overexpression in HSFs increased levels of Col 1A1, Col 1A2, α-SMA, and CTGF transcript, and miR-16-5p inhibition partially reversed these increases (Figure 7C–7F). Furthermore, MMP-1 and MMP-8 expression decreased when NOTCH2 was overexpressed, and inhibition of miR-16-5p partially restored MMP-1 and MMP-8 levels (Figure 7G–7H).

## DISCUSSION

Myofibroblasts play a key role in tissue fibrosis, and α-SMA expression is a significant marker of myofibroblast activation [19]. The proportion of myofibroblasts in the overall fibroblast population is closely associated with the severity of fibrosis-related diseases [20]. For an example, a recent study indicated that α-SMA expression was increased in fibroblasts cultured from SSc skin biopsies, which is consistent with the transforming growth factor-β (TGF-β)-induced increase in α-SMA levels observed *in vitro*. Furthermore, a recent single-cell RNA-sequencing analysis identified different subgroups of fibroblasts in human skin samples [21]. In this study, we found that α-SMA levels were elevated both in antagomiR-16-5p-treated and NOTCH2 overexpression plasmid-treated HSFs, suggesting that both miR-16-5p inhibition and NOTCH2 overexpression can activate myofibroblasts in HSFs.

Our bioinformatics analyses also indicated that miR-16-5p is a regulatory factor for priming myofibroblasts. miR-16-5p expression was reduced in SSc patients compared to healthy volunteers, and miR-16-5p knockdown induced α-SMA expression in HSFs. Additionally, miR-16-5p has previously been reported to regulate epigenetic changes in connective tissue and to play a key role in immune diseases [11]. We therefore hypothesized that miR-16-5p expression would affect collagen secretion in HSFs and performed additional *in vitro* and *in vivo* assays to confirm the role of miR-16-5p in fibrosis.

Matrix metallopeptidases (MMPs) are known to degrade a variety of extra cellular matrix components and play significant roles in tissue fibrosis processes [22]. The collagenases MMP1 and MMP8 initiate the cleavage of fibrillar collagen, the digested products of which are further degraded by other MMPs [23]. In this study, we found that inhibition of miR-16-5p reduced MMP-1 and MMP-8 expression in HSFs, indicating that miR-16-5p has direct antifibrotic effects. However, the potential molecular mechanisms underlying those effects should be investigated further in future studies.

Prior studies reported that NOTCH signaling, which is responsible for profibrotic phenotypes *in vitro* and tissue fibrosis *in vivo*, is activated in fibroblasts extracted from SSc patients’ skin [24]. Accumulating evidence also indicates that miR-16-5p is an important regulator of NOTCH signaling [25–26]. The bioinformatics results of this study indicate that NOTCH2 is enriched in SSc patients, and qRT-PCR also demonstrated that NOTCH2 levels were elevated in the serum of SSc patients. We therefore verified the association between miR-16-5p and NOTCH2 *in vitro* and found that miR-16-5p partially reversed NOTCH2-dependent myofibroblast activation. These results suggest that miR-16-5p affects myofibroblast activation by regulating NOTCH signaling.

In summary, our data show that miR-16-5p is downregulated in SSc patients compared to healthy controls, and this downregulation is crucial for NOTCH2 signaling-induced myofibroblast activation. Additionally, our findings suggest that drugs that inhibit miR-16-5p overexpression might be a promising clinical treatment for SSc.

## MATERIALS AND METHODS

### Identification of differentially expressed genes (DEGs) and differentially expressed miRNAs (DEMs)

The GSE137472 (miRNAs) and GSE145120 (mRNAs) microarray datasets including SSc patients and control subjects were retrieved from NCBI Gene Expression Omnibus (GEO). The online software GEO2R was used to identify DEGs using default settings. Gene expression was visualized using imageGP (http://www.ehbio.com/ImageGP/index.php/home/index/pheatmap.html). Genes with logFC >0.4 or <-0.4 were considered DEGs, and miRNAs with logFC >0.1 or <-0.1 were considered DEMs.

### Gene ontology (GO) and kyoto encyclopedia of genes and genomes (KEGG) pathway analyses

The online Database for Annotation, Visualization and Integrated Discovery (DAVID) Bioinformatics Resources version 6.8 tool was used to perform GO term and KEGG pathway enrichment analysis of the DEGs. Results were visualized by GOplot, and the volcano plot was generated using GraphPad Prism 8.0.

### MiRNA target gene prediction

The online prediction tool TargetScanHuman (http://www.targetscan.org/vert_72/) was used to identify target genes of the differentially expressed miRNAs. The results of target gene analysis were visualized using Cytoscape 3.7.2.

### Ethical statement

The study protocols were approved by the Ethics Committee of Affiliated Zhoupu Hospital, Shanghai University of Medicine and Health Sciences, and informed consent was obtained from all participants.

### Blood collection

Peripheral blood samples were collected from patients at our Hospital. (30 healthy volunteers, 30 SSc patients) from May 2018 to March 2020 for measurement of miR-16-5p and NOTCH2 mRNA expression.

### Cell culture and transfection

High-glucose Dulbecco's modified eagle medium (Gibco BRL, Grand Island, USA) was used to culture human skin fibroblasts (HSFs, FuHeng Biology, Shanghai, China). HSFs were cultured at 37° C with 5% CO_2_ and 95% humidity. The agomiR and antagomiR transfection constructs were purchased from GenePharma (Shanghai), and transfection was performed according to the manufacturer’s protocol using a concentration of 200 mM. RNA interference was used for endogenous NOTCH2 knockdown. siRNA oligonucleotides were purchased from Life Technologies, and transfection was performed using Lipofectamine RNAiMAX (Life Technologies) according to the manufacturer's protocol. The NOTCH2 expression plasmid was purchased from the Public Protein/Plasmid Library (PPL, #BC071562).

### Luciferase reporter assay

The putative miR-16-5p target site (nucleotides 44-50) was identified using TargetScan (version 7.0; http://www.targetscan.org/vert_70/). PCR was then used to amplify this target site from HSF cDNA, and the sequence was ligated to the pGL3-basic vector (Promega Corporation). To create the pGL3-*NOTCH2*-3’UTR-mutant (Mut) vector, two site mutations were made in the potential miR-16-5p target site using a QuikChange Site-Directed Mutagenesis kit (Agilent Technologies, Inc.). Next, Lipofectamine® 2000 (Thermo Fisher Scientific) was used to co-infect HSFs with the *Renilla* plasmid and either pGL3-*NOTCH2*-3’UTR-wild-type (200 ng) or pGL3-*NOTCH2*-3’UTR-Mut (200 ng). HSFs were then transfected with the miRNA negative control mimic (10 nM) or miR-16-5p mimic (10 nM) for 48 h at 37° C. A Dual-Luciferase Reporter assay system (Promega Corporation) was used to measure the relative luciferase activity of each well.

### qRT-PCR analysis

First, total RNA was isolated from cells and serum samples using TRIzol® reagent (Thermo Fisher Scientific). Next, the reverse-transcription was performed using ReverTra Ace® qPCR RT Master Mix (Toyobo Life Science) to obtain cDNA based on the manufacturer’s protocol. Relative miRNA levels were normalized to *GAPDH* levels (the internal control) and were calculated using the 2^-ΔΔCt^ approach. All experiments were conducted in triplicate; miRNA and mRNA primer sequences are listed in Table 3.

**Table 3:** microRNAs and mRNA primer sequences.

**microRNA or gene names**	**Primer sequence (5’ to 3’)**
hsa-miR-16-5p-Forward	TGGGGTAGCAGCACGTAAA
hsa-miR-16-5p-Reverse	CTCAACTGGTGTCGTGGAGTC
hsa-U6-Forward	CTCGCTTCGGCAGCACA
hsa-U6-Reverse	AACGCTTCACGAATTTGCGT
hsa-MMP-1-Forward	CTGCTTACGAATTTGCCGACAGA
hsa-MMP-1-Reverse	GTTCTAGGGAAGCCAAAGGAGCTG
hsa-MMP-8-Forward	AGTGCCTGACAGTGGTGGTT
hsa-MMP-8-Reverse	TCCCTGTGAGATCCTGGTGA
hsa-Notch2-Forward	GGGACCCTGTCATACCCTCT
hsa-Notch2-Reverse	GAGCCATGCTTACGCTTTCG
hsa-α-SMA-Forward	TGTATGTGGCTATCCAGGCG
hsa-α-SMA-Reverse	AGAGTCCAGCACGATGCCAG
hsa-Col-1a1-Forward	CCTCCAGGGCTCCAACGAG
hsa-Col-1a1-Reverse	TCTATCACTGTCTTGCCCCA)
hsa-Col-1a2-Forward	GATGTTGAACTTGTTGCTGAGC
hsa-Col-1a2-Reverse	TCTTTCCCCATTCATTTGTCTT
hsa-CTGF-Forward	GTGTGCACTGCCAAAGATGGT
hsa-CTGF-Reverse	TTGGAAGGACTCACCGCT
hsa-GAPDH-Forward	ACCCACTCCTCCACCTTTGA
hsa-GAPDH-Reverse	CTGTTGCTGTAGCCAAATTCGT

### Western blotting

Total protein was extracted from HSFs or blood samples, resolved using SDS-PAGE gels (10-15% TrisGlycine), transferred onto Hybond nitrocellulose membranes (Amersham Biosciences), and probed with antibodies specific for NOTCH2 (1:500, #ab8927, Abcam, Cambridge, UK) and GAPDH (1:1000, #ab8245, Abcam). Immunoblots were visualized using species-specific HRP conjugated secondary antibodies (Abcam) and electrochemiluminescence (Thermo/Pierce) on a Bio-Rad ChemiDoc imaging system.

### Statistical analysis

GraphPad Prism 8.0 was used to perform statistical analyses. Results are shown as mean ± SD. Student’s t-tests were used to compare two groups, while one-way analysis of variance with Tukey’s post hoc test was used to compare more than two groups. A significance threshold of *p* < 0.05 was used.

### Ethical approval

The Ethics Committee of The Affiliated Zhoupu Hospital, Shanghai University of Medicine and Health Sciences, Shanghai 201318, China approved this study.

## Supplementary Material

Supplementary Figures
