# Supplementary material for: MiR-16-5p suppresses myofibroblast activation in systemic sclerosis by inhibiting NOTCH signaling
Source: Aging (Albany NY). 2020 Dec 19;13(2):2640–54. doi: 10.18632/aging.202308 (PMC7880343; doi:10.18632/aging.202308)
Supplement: Supplementary Figures [file aging-13-202308-s001.pdf]

SUPPLEMENTARY FIGURES

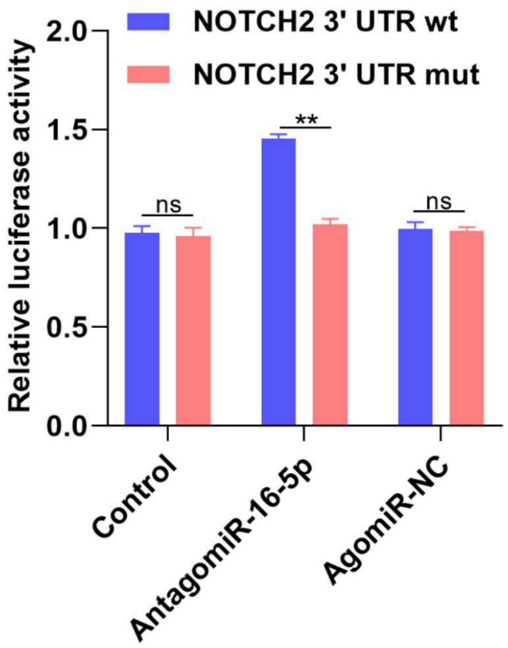

Supplementary Figure 1. The relation of miR-16-5p and NOTCH2 was verified by luciferase assays.

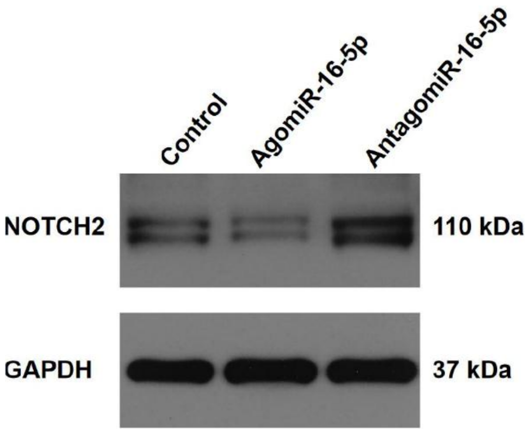

Supplementary Figure 2. Western blot result of NOTCH2 in the different groups.

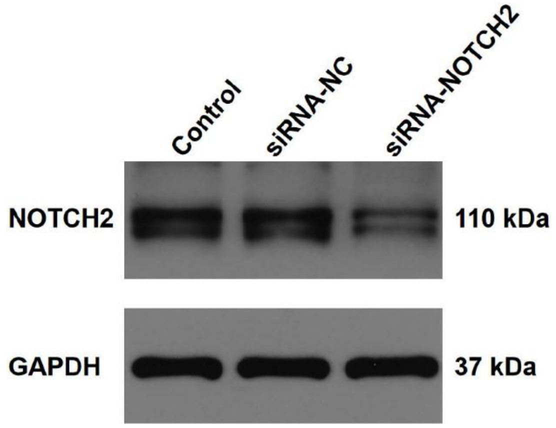

Supplementary Figure 3. Western blot result of NOTCH2 in the different groups.

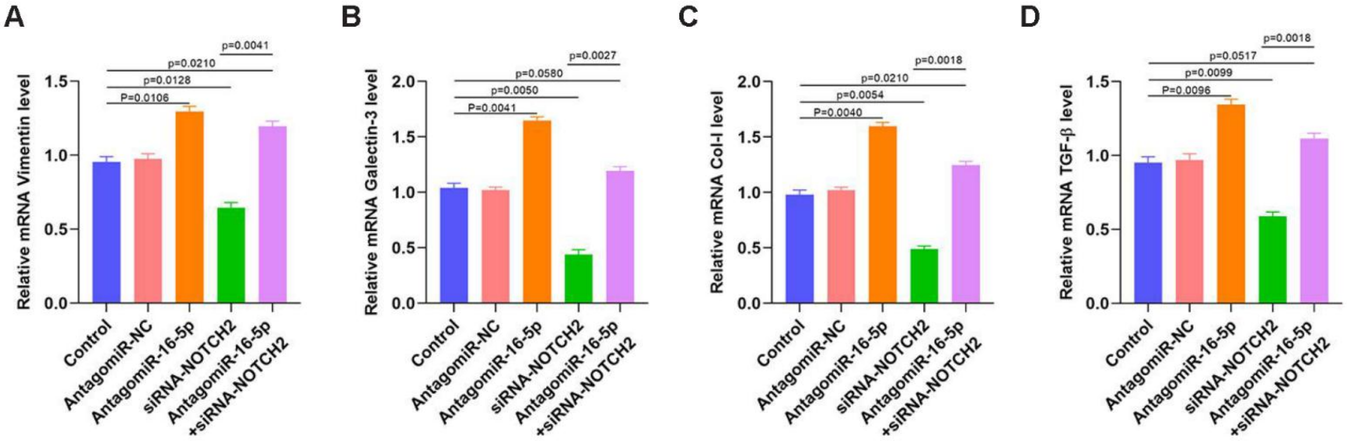

Supplementary Figure 4. Inhibition of miR-16-5p activates myofibroblasts. (**A**) The level of Vimentin in different groups was measured by qRT-PCR analysis. (**B**) The level of Galectin-3 in different groups was measured by qRT-PCR analysis. (**C**) The level of Col-1 in different groups was measured by qRT-PCR analysis. (**D**) The level of TGF-β in different groups was measured by qRT-PCR analysis.

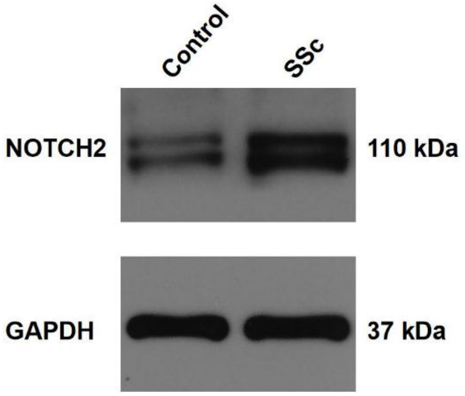

Supplementary Figure 5. Western blot result of NOTCH2 in the different groups.

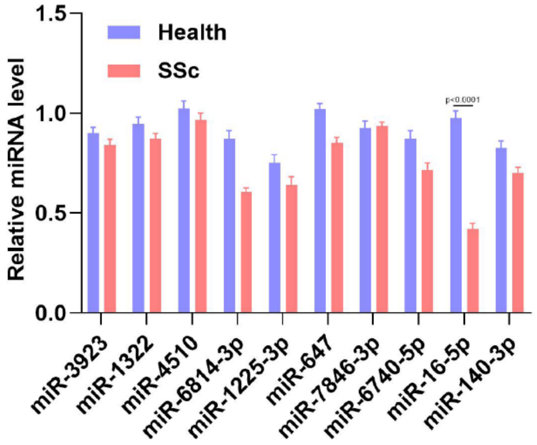

Supplementary Figure 6. qRT-PCR results of miRNAs in the different groups.

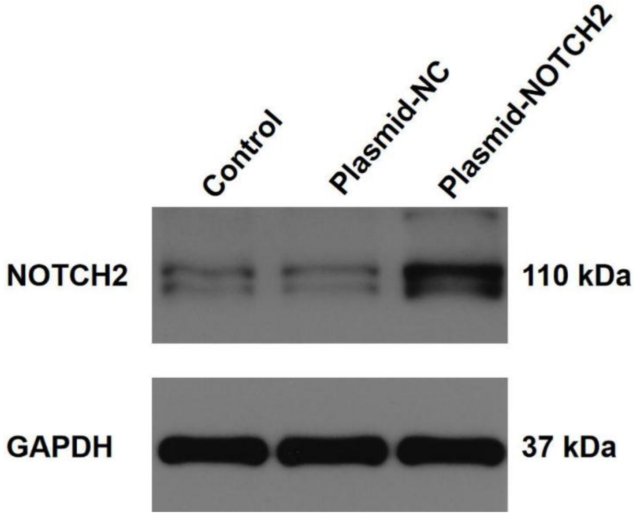

Supplementary Figure 7. Western blot result of NOTCH2 in the different groups.
